# Transients drive the demographic dynamics of plant populations in variable environments

**DOI:** 10.1111/1365-2745.12528

**Published:** 2016-02-22

**Authors:** Jenni L. McDonald, Iain Stott, Stuart Townley, Dave J. Hodgson

**Affiliations:** ^1^ Centre for Ecology and ConservationCollege of Life and Environmental SciencesUniversity of ExeterPenrynTR10 9FEUK; ^2^ Max Planck Institute for Demographic ResearchKonrad‐Zuse‐Straße 1RostockDE‐18057Germany; ^3^ Max Planck Odense Center on the Biodemography of AgingUniversity of Southern DenmarkCampusvej 55OdenseDK‐5230Denmark; ^4^ Environment and Sustainability InstituteUniversity of ExeterPenrynTR10 9FEUK

**Keywords:** asymptotic dynamics, demography, environmental stochasticity, matrix population models, plant population dynamics, stochastic, transient dynamics

## Abstract

1. The dynamics of structured plant populations in variable environments can be decomposed into the ‘asymptotic’ growth contributed by vital rates, and ‘transient’ growth caused by deviation from stable stage structure.
2. We apply this framework to a large, global data base of longitudinal studies of projection matrix models for plant populations. We ask, what is the relative contribution of transient boom and bust to the dynamic trajectories of plant populations in stochastic environments? Is this contribution patterned by phylogeny, growth form or the number of life stages per population and per species?
3. We show that transients contribute nearly 50% or more to the resulting trajectories, depending on whether transient and stable contributions are partitioned according to their absolute or net contribution to population dynamics.
4. Both transient contributions and asymptotic contributions are influenced heavily by the number of life stages modelled. We discuss whether the drivers of transients should be considered real ecological phenomena, or artefacts of study design and modelling strategy. We find no evidence for phylogenetic signal in the contribution of transients to stochastic growth, nor clear patterns related to growth form. We find a surprising tendency for plant populations to boom rather than bust in response to temporal changes in vital rates and that stochastic growth rates increase with increasing tendency to boom.
5. *Synthesis*. Transient dynamics contribute significantly to stochastic population dynamics but are often overlooked in ecological and evolutionary studies that employ stochastic analyses. Better understanding of transient responses to fluctuating population structure will yield better management strategies for plant populations, and better grasp of evolutionary dynamics in the real world.

## Introduction

Plants persist and evolve in variable environments. As a result, their population abundances fluctuate through time. Understanding the drivers of these fluctuations may be key to an improved understanding of life‐history evolution (Tuljapurkar 1982, 1989; Tuljapurkar, Gaillard & Coulson 2009; Rees & Ellner 2016), conservation of endangered plant species (Smith, Caswell & Mettler‐Cherry 2005; Coates, Lunt & Tremblay 2006; Pfeifer *et al*. 2006), sustainable exploitation of harvestable species (Gaoue & Ticktin 2010), control of weeds (Parker 2000; Holst, Rasmussen & Bastiaans 2007) and management of invasive species (Sebert‐Cuvillier *et al*. 2007; Kerr *et al*. 2016). Classic approaches to studying stochastic demography consider schedules of survival, growth and reproduction, collectively termed ‘vital rates’, as random variables. Variation in vital rates may be determined by both exogenous drivers (e.g. weather, nutrient availability or interspecific competition) and/or endogenous drivers (e.g. demographic stochasticity or intraspecific competition). Vital rate variation may be observed under specific conditions at different points in time or space, or modelled using random draws from distributions of potential vital rates (Fieberg & Ellner 2000). This yields stochastic analyses that work with long‐run expectations of population growth or decline (Tuljapurkar, Gaillard & Coulson 2009).

Recent advances have revealed that noisy stochastic population dynamics can be decomposed into two parts. The first component describes the influence of asymptotically stable growth or decline. In a stable environment, population structure and growth rate are expected not to change over time: the distribution of ages or stages is expected to remain at a stable structure, with an associated stable growth rate of the population (Caswell 1978; Hodgson & Townley 2004). In unstable environments, disturbances to population structure and perturbations to vital rates create a mismatch between actual population structure and stable population structure. This causes ‘transient dynamics’: short‐term, non‐stable fluctuations in population growth and density, which are different to stable growth (Stott, Townley & Hodgson 2011; Ellis & Crone 2013). Transient dynamics are the second component of stochastic population dynamics (Ellis & Crone 2013), and their nature depends on population structure.

All plant life histories are structured in some way: vital rates depend on the age, size, life stage, health, or other status of an individual. This structuring means that not all individuals contribute equally to population dynamics: an overrepresentation of reproductive individuals results in transient growth that is faster than the stable growth rate, while a bias towards individuals of low reproductive value (e.g. immature individuals) causes transient growth that is slower than the stable growth rate. Thus in the short term, populations with non‐stable stage structures will either grow faster (or decline slower) than the stable growth rate (termed ‘amplification’ or ‘boom’), or will grow slower (or decline faster) than the stable growth rate [termed ‘attenuation’ or ‘bust’; (Stott *et al*. 2010a)].

Assumptions of population stability are rarely applicable to species suffering from disturbance or perturbation, so transient effects are probably common in the natural world (Ezard *et al*. 2010). Ignoring non‐stable population structure may result in the failure of management efforts, as short‐term population dynamics can differ considerably from stable growth rate (Koons *et al*. 2005). For example, the cessation of harvesting of heavily exploited fish populations naturally perturbs populations away from stable equilibriums, resulting in long transient periods and changes in abundance which deviate from the expected long‐term growth rate (White *et al*. 2013). If populations experience frequent disturbances which change their structure and reduce overall density, such as disease epidemics (Needham *et al*. 2016), fire (Treurnicht *et al*. 2016), or extreme weather events (Uriarte *et al*. 2016), transient amplification will help rebound from reduced density. Phenotypes which are able to amplify their number in response to such disturbances should have a competitive advantage over ones that do not: thus, it is possible that these phenotypes should be selected for. Transient dynamics are, however, largely ignored in ecological and evolutionary studies in favour of stable, equilibrium or long‐term measures. If transient dynamics comprise a large component of stochastic dynamics, then they deserve explicit attention in studies that employ demography to inform population management, or which address ecological and evolutionary questions.

In recognition of this, methods for the study of transient dynamics have flourished (Hastings 2004; Koons *et al*. 2005; Townley *et al*. 2007; Townley & Hodgson 2008; Tenhumberg, Tyre & Rebarber 2009), and the use of transient dynamics in population management is on the rise (Koons, Rockwell & Grand 2006; Ezard *et al*. 2010; Ticktin *et al*. 2012; Baines, Eager & Jarosz 2014; Tremblay, Raventos & Ackerman 2015). Our goal here is to study the relative contributions of transient dynamics and stable dynamics to stochastic population dynamics, across 277 populations of 132 species of plants whose demographies have been measured across multiple years. This extends the study of Ellis & Crone (2013), who concluded that across nine species of herbaceous perennial, transients account for > 50% of variation in stochastic time series and that transients generally buffer the effects of asymptotic growth. Our study extends the breadth of these data, which is made possible by the advent of the compadre global data base of projection matrix models for plant populations (Salguero‐Gomez *et al*. 2015), and the methodology, as we introduce a new measure that recognizes potential antagonism in the influences of stable vs. transient dynamics.

## Materials and methods

### Stochastic Projection Matrix Models

A standard approach to modelling structured demographic dynamics is to represent the vital rates as a projection matrix (Caswell 2014), which projects a structured vector of stage abundances (**x**) through time (*t*). Entries in the projection matrix [**A**: a square, non‐negative, generally primitive and irreducible matrix; (Stott *et al*. 2010b)] describe rates at which members of each stage class at time *t* remain in their stage class or move to a new stage class at time *t+*1:

(eqn 1) $$x\left(t+1\right)=\mathrm{Ax}\left(t\right)$$

In stable environments, in the absence of density‐ or resource‐dependent effects, the asymptotically stable growth rate of the population described by (eqn 1) is the dominant eigenvalue of **A** (λ_*max*_: the eigenvalue with maximum absolute value, which is generally real and positive; (Caswell 2014). The stable stage structure is its associated right eigenvector (**w**). However, the current stage structure is usually not proportional to the stable stage structure (i.e. **x**(*t*) is not proportional to **w**; (Williams *et al*. 2011), and vital rates of the projection matrix vary over time (**A** becomes **A**(*t*) as the entries of **A** change with *t*; Fieberg & Ellner (2001)). In this case, population growth or decline between *t* and *t+*1 can be decomposed as:

(eqn 2) $$\frac{{∥x(t+1)∥}_{1}}{{∥x(t)∥}_{1}}\;\;=\lambda_{obs}\left(t\right)=\lambda_{max}\left(t\right)\cdot reactivity\;\left(t\right)$$

where λ_*obs*_(*t*) is the observed growth rate between time *t* and time *t+*1, λ_*max*_(*t*) is the dominant eigenvalue of **A**(*t*) and ║**x**║_1_ denotes the sum, or ‘one‐norm’, of elements in any column vector **x**. The right‐hand side of the product in (eqn 2) is a standard measure of single‐timestep transient amplification or attenuation, called *reactivity* (Neubert & Caswell 1997; Townley *et al*. 2007; Townley & Hodgson 2008). It measures the instantaneous boom or bust as a result of deviation of current stage structure from stable stage structure and is calculated as:

(eqn 3) $$reactivity\;\left(t\right)={∥\frac{A(t)}{\lambda_{max}\left(t\right)}\cdot \frac{x(t)}{{∥x(t)∥}_{1}}∥}_{1}$$

It is convenient and standard to log‐transform these multiplicative dynamics to make them additive, such that:

(eqn 4) $$log\left(\lambda_{obs}\left(t\right)\right)=log\left(\lambda_{max}\left(t\right)\right)+log\left(reactivity\left(t\right)\right)$$

This decomposition of per‐timestep dynamics reveals that at any moment in time, asymptotically stable dynamics encourage populations to grow or decline, while transients either amplify or attenuate that growth or decline. Hence, transients can either exaggerate or buffer the ‘current’ asymptotic growth rate.

**Figure 1:**
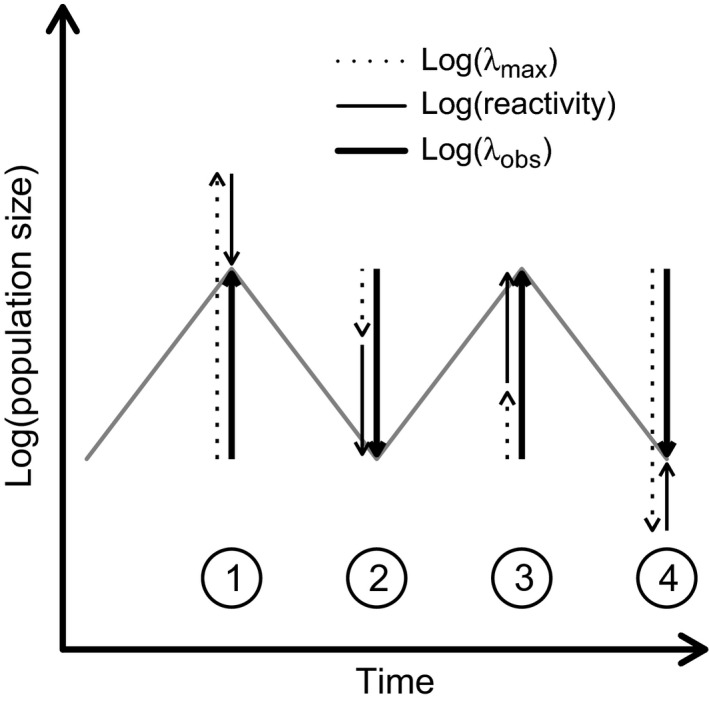
The partitioning of changes in population size into the contributions due to transients (log (*reactivity*)) and asymptotic growth (log (λ_*max*_)). Fluctuations 1 and 3 show identical rates of growth (log (λ_*obs*_)) and 2 and 4 show identical declines; however, their underlying dynamics differ. Transients can either exaggerate the asymptotic growth rate (2 & 3), or act in opposition to asymptotic growth/decline generating a buffering effect (1 & 4). The underlying absolute dynamics for the first scenario are thus weaker than those where asymptotic and transient growths have opposite signs.

Equation 4 calculates the net impact of asymptotic growth and reactivity on observed rates of increase, but this approach hides what we call the underlying *absolute dynamic* (λ_*abs*_). When asymptotic and transient growth have opposite signs, strong but opposing forces could yield little change in observed growth; this should be differentiated from weak forces of growth and amplification or attenuation (Fig. 1). We can calculate the underlying absolute dynamic using:

(eqn 5) $$log\left(\lambda_{abs}\left(t\right)\right)=|log\left(\lambda \left(t\right)\right)|+|log\left(reactivity\left(t\right)\right)$$

where |*x*| represents the absolute value of the scalar *x*.

### Data

Our matrix projection models come from the compadre data base (Salguero‐Gomez *et al*. 2015). We chose plant populations in the compadre data base (version 3.0) that satisfied three criteria: they should be represented by at least three time‐dependent projection matrices; they should not be affected by experimental manipulation; they should not be the averages of multiple matrices. In total, this comprises 277 populations of plants for 132 species. We excluded two species from this data set as they were sole representatives of their life history (the alga *Gracilaria gracilis* and the epiphyte *Tillandsia brachycaulos*), and two fern species that they represented only a single genus and made up all ferns in our sample (*Asplenium adulterinum* and *A. cuneifolium*). The remaining studies are naturally biased towards herbaceous perennials and also contain considerable numbers of populations for other life forms including shrubs, succulents, palms and trees.

### Metrics of Growth

We follow Ellis & Crone (2013) by simulating the dynamics of each plant population, starting with equal numbers of individuals in each life stage and drawing projection matrices at random with equal probability from the available set of matrices. We run 1000 simulations per population, giving each a burn‐in of 500 time intervals to remove any influence of starting conditions. Between *t = *500 and *t = *501, we calculate λ_*max*_ as the dominant eigenvalue of **A**(500), *reactivity* using (eqn 3) (Stott, Hodgson & Townley 2012b), λ_*obs*_ using (eqn 2) and λ_*abs*_ using (eqn 5), yielding 1000 measures of each metric per population for analysis.

### Partitioning Dynamics

Ellis & Crone (2013) performed replicate simulations of stochastic dynamics and decomposed growth after burn‐in log(λ_*obs*_) (which they term ‘*r*_*obs*_’) into its non‐independent contributors log(λ_*max*_) (which they term ‘*r*_*VR*_’) and log(*reactivity*) (which they term ‘*r*_*TD*_’). We follow Ellis & Crone (2013) in calculating, for each population of each species, the proportion of variance in log(λ_*obs*_) due to variation in log(λ_*max*_) or due to variation in log(*reactivity*). This is equal to the square of the Pearson's correlation coefficient between the 1000 measures per population of log(λ_*obs*_) and log(λ_*max*_), and log(λ_*obs*_) and log(*reactivity*), respectively. As log(λ_*obs*_) is a net measure of their influence, these R^2^ values need not sum to 1. We add to this by calculating the proportion of absolute dynamics that is due to transients using |log(*reactivity*)|/ log(λ_*abs*_). As each simulation yields an independent value of this metric, we calculated its mean across the 1000 simulation replicates for each population of each species to yield one per population for analysis.

### Statistical Methods

Our primary goal was to determine simply how much contribution transients make to stochastic dynamics, irrespective of modelling strategy or plant traits. However, we also study the influence of ecological, evolutionary and modelling parameters on the contribution of transients. All statistics were done using MCMCglmm (Hadfield 2010) in R3.1.2 (R Core Team 2014). Models were run for 500000 iterations with a 10% burn‐in and thinning interval of 250, to give effective sample sizes of around 2000 per parameter per model. Proper diffuse priors were used for fixed effects (normal distribution with μ = 0, σ^2 ^= 10^10^), parameter‐expanded proper diffuse priors for random effects (inverse Wishart distribution with V = 1 and ν = 0.001, alpha‐μ = 0 and alpha‐V = 100), and proper diffuse priors for residuals (inverse Wishart distribution with V = 1 and ν = 0.001). Chains mixed well and showed low autocorrelations of < 0.1.

Our response variables were as follows: (i) amount of observed growth variation explained by asymptotics [square of the Pearson's correlation coefficient between log(λ_*obs*_) and log(λ_*max*_)]; (ii) amount of observed growth variation explained by transients [square of the Pearson's correlation coefficient between log(λ_*obs*_) and log(*reactivity*)]; (iii) proportion of absolute dynamics due to transients [|log(*reactivity*)|/log(λ_*abs*_)]; (iv) stochastic growth rate [mean log(λ_*obs*_) per population across all simulations]; (v) mean asymptotic growth rate [mean log(λ_*max*_) per population across all simulations]; and (vi) mean transient growth [mean log(*reactivity*) per population across all simulations]. Measures 1–3 are proportions and so are bounded between 0 and 1. To prevent the algorithm fitting values outside these bounds, we logit‐transformed these variables prior to analysis.

Our fixed explanatory variables for each model were plant growth form, as described in compadre (including herbaceous perennials, shrubs, succulents, palms and trees) and the number of life stages in the projection matrix. We consider a fixed parameter (a slope or intercept) to be ‘important’ or ‘identifiable’ if its 95% credible intervals do not overlap zero, or when comparing between different parameters, if the 95% credible intervals do not overlap the mean for other parameters in the group.

A random intercept for species was included in all models, as multiple populations of many species are present in our data sample. We also tested the importance of shared ancestry by scaling random effects by the inverse variance–covariance distance matrix estimated by the phylogeny associated with our species (Salguero‐Gomez *et al*. 2015). For every phylogenetic model, posterior variance distributions for the effect of phylogeny were not different from zero, and the relative variance attributable to phylogeny, calculated using the ratio of phylogenetic variance to the sum of phylogenetic variance, species variance and residual variance (Hadfield & Nakagawa 2010), was low (see Appendix S1 in Supporting Information). We concluded that the variables used in analyses showed no phylogenetic signal, and results reported in this manuscript are for models excluding phylogenetic relationships.

## Results

### Contribution of Asymptotics to Net Stochastic Growth

**Figure 2:**
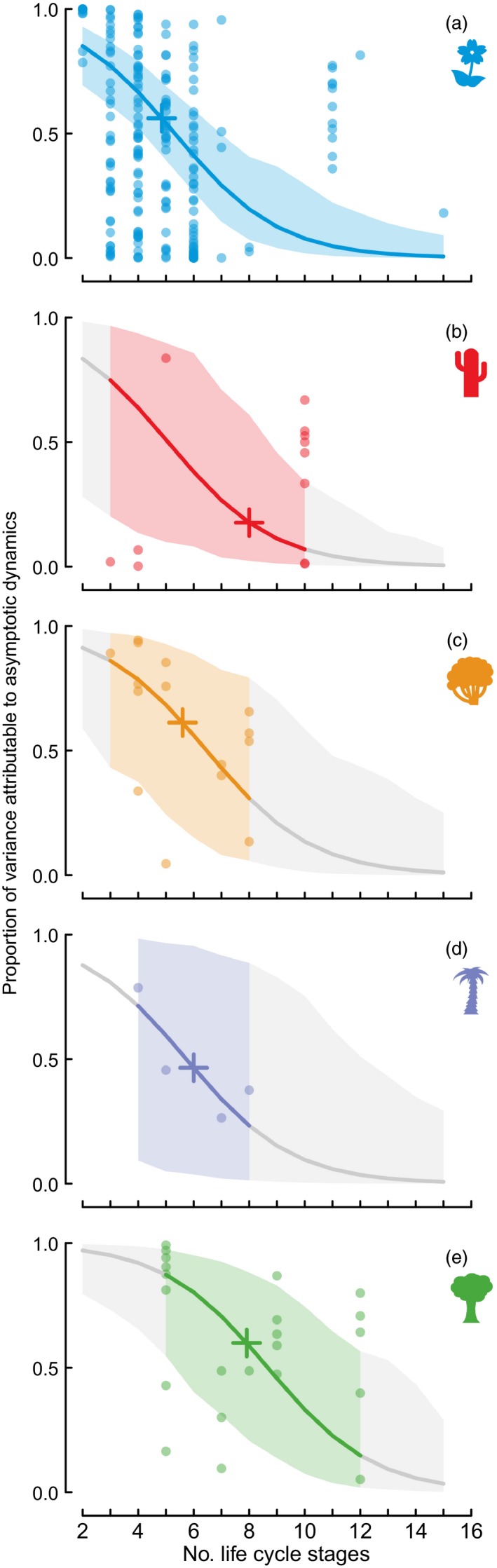
Proportion of variation in net growth (log (λ_*obs*_)) attributable to asymptotic dynamics (log (λ_*max*_)) decreases with matrix dimension: (a) herbaceous perennials, (b) succulents, (c) shrubs, (d) palms and (e) trees. Coloured lines and shading indicate fitted relationships and 95% credible intervals within the data range; grey lines and shading indicate these values outside the range. Plus signs indicate predicted values the mean matrix dimension of each growth form. Results plotted are from a model with fixed effects of matrix dimension and growth form, and a random effect of species. Credible intervals indicate no detectable differences between growth forms, but matrix dimension is an important effect whether growth form is included in the model or not.

Variation in asymptotics explains on average 47.8 ± 2.0% of the variance in stochastic growth rates (raw mean ± standard error). The contribution of asymptotics to net stochastic growth varied among growth forms, with trees having higher proportions by asymptotics than the other life forms, although 95% credible intervals indicated that life form is not statistically important (Fig. 2). Larger matrix dimensions were associated with identifiably smaller variance explained by asymptotics (mean slope with 95% credible intervals on logit scale of −0.53 ± 0.23; Fig. 2).

### Contribution of Transients to Net Stochastic Growth

**Figure 3:**
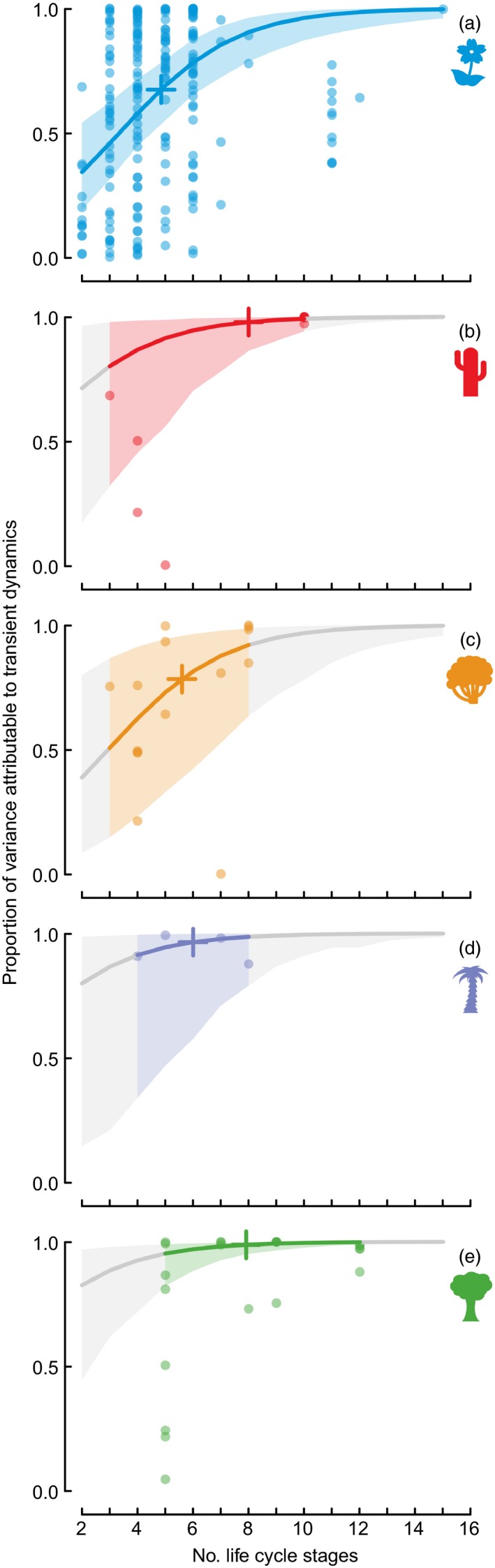
Proportion of variation in net growth (log (λ_*obs*_)) attributable to transient dynamics (log (*reactivity*)) increases with matrix dimension: (a) herbaceous perennials, (b) succulents, (c) shrubs, (d) palms and (e) trees. Coloured lines and shading indicate fitted relationships and 95% credible intervals within the data range; grey lines and shading indicate these values outside the range. Plus signs indicate predicted values the mean matrix dimension of each growth form. Results plotted are from a model with fixed effects of matrix dimension and growth form, and a random effect of species. Credible intervals indicate no detectable differences between growth forms, but matrix dimension is an important effect whether growth form is included in the model or not.

Variation in reactivity explains on average 64.8 ± 2.0% of the variance in stochastic growth rates (raw mean ± standard error). The amount of variation explained by reactivity was different among different life forms, with trees, palms and succulents having relatively more of their dynamics explained than shrubs and herbs, although again 95% credible intervals indicated this not to be statistically important (Fig. 3). The number of life stages had an important influence, with proportion of variance in observed growth increasing with increasing matrix dimension (mean slope with 95% credible intervals on logit scale of 0.48 ± 0.23; Fig. 3).

### Contribution of Transients to Absolute Dynamics

**Figure 4:**
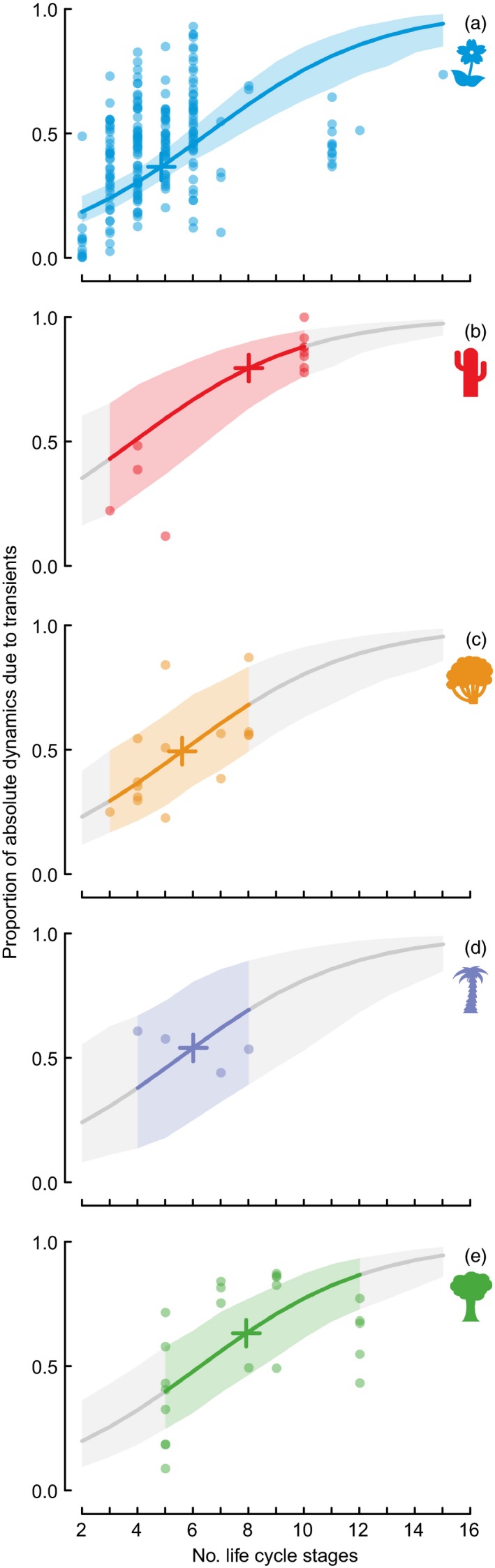
Proportion of absolute dynamics (log (λ_*abs*_)) due to transient dynamics (log (*reactivity*)) increases with matrix dimension and differs for (a) herbaceous perennials, (b) succulents, (c) shrubs, (d) palms and (e) trees. Coloured lines and shading indicate fitted relationships and 95% credible intervals within the data range; grey lines and shading indicate these values outside the range. Plus signs indicate predicted values the mean matrix dimension of each growth form. Results plotted are from a model with fixed effects of matrix dimension and growth form, and a random effect of species. Credible intervals indicate no detectable differences between growth forms, but matrix dimension is an important effect, whether growth form is included in the model or not.

We measured the mean proportion of absolute dynamics that were due to reactivity, per population, per species. On average reactivity contributed 46.6 ± 1.3% of the underlying dynamics (raw mean ± standard error). This varied among life forms (Fig. 4), although for the mean average matrix dimension of 5.3 was distributed around 50%. The 95% credible intervals again showed a lack of statistical importance for life form. Larger matrices had identifiably more of their absolute dynamics contributed from transients (mean slope with 95% credible intervals of 0.33 ± 0.10; Fig. 4). We note that the mean proportion of underlying dynamics due to asymptotics is the complement of that due to reactivity, and so is not analysed here.

### An Overall Tendency to Boom

**Figure 5:**
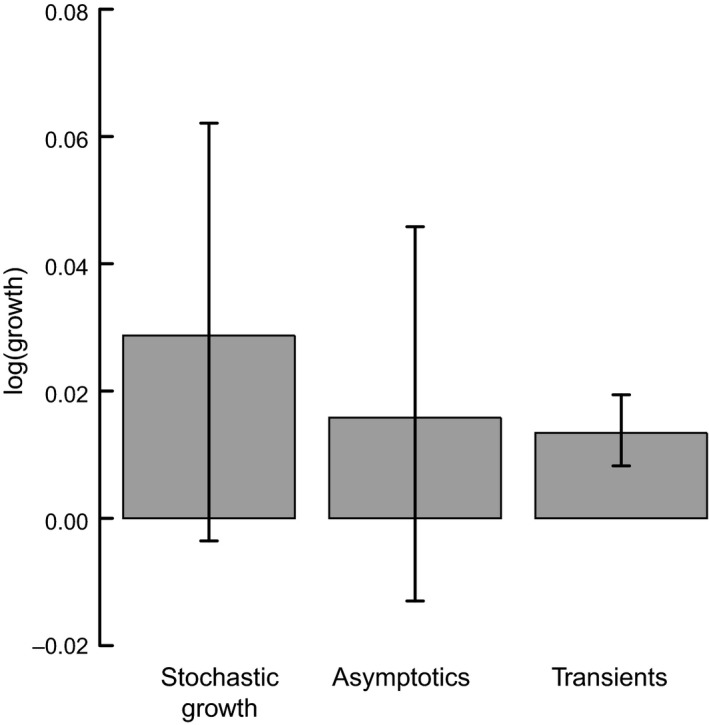
The log (growth rates) alongside 95% credible intervals for the observed stochastic growth rate and the growth rates that can be attributed to asymptotics and transients. Results plotted are from three separate models with null fixed effects and a random effect of species.

Our method allows us to study the overall tendency of plant populations to boom or bust in response to time‐varying stable stage structure. The null hypothesis prediction is that mean reactivity across all simulations should be zero: populations should be equally likely to bust as to boom in response to fluctuations in vital rates. Such is not the case. In fact, global mean reactivity predicted by the null model without any fixed effects was 0.013 (95% credible intervals ±0.0055), which is identifiably different from zero (pMCMC = 0.019, Fig. 5). This tendency to boom was not influenced by any explanatory variables described above. Conversely, mean global λ_obs_ and mean global λ_max_ were not identifiably different from 0 according to their null models (Fig. 5). Indeed, the distribution of λ_max_ across all matrices within the focal populations is clearly distributed around zero (Fig. S1). Linear mixed effects models (with random effects of species and population) did show significant positive relationships between mean log‐reactivity and log‐stochastic growth rate (χ^2^_1 _= 76.626, *P *≪ 0.001), and mean log‐reactivity and log‐stable growth rate (of the mean projection matrix for each population of each species; χ^2^_1_ = 76.633, *P *≪ 0.001).

## Discussion

Transients clearly play an important role in shaping the population trajectories of plants in variable environments. Populations will react to stochastic changes in vital rates by booming or busting. This can exaggerate or buffer the expected asymptotic growth or decline as predicted by current vital rates. When measured as a non‐independent contribution to net stochastic growth, transients are responsible, on average, for more than half of variation in observed growth. When measured as an independent contribution, including the combined push and pull of asymptotic and transient effects, transients still explain nearly half of the total absolute dynamic. These results echo those found in 9 species of perennial herb by Ellis & Crone (2013), showing that the contribution of transients to stochastic dynamics is large and consistently important across a very wide range of plant species. We found evidence that the contribution of transients to stochastic growth may depend on species‐level traits (life‐history complexity) and on modelling artefacts (number of life stages modelled). Unexpectedly, we found an overall tendency for reactivity to be positive, and a significant positive driver of long‐term stochastic growth rates.

### Contribution of Asymptotics to Net Dynamics

Stochastic environments drive temporal variation in vital rates, resulting in fluctuations in the expected asymptotic growth rate over time. It is satisfying to find that this time‐varying asymptotic measure of population growth explains almost exactly half (48%) of the variation in observed growth rate across stochastic population trajectories. The contribution of asymptotic growth to stochastic dynamics decreases with the number of life stages, whether or not these are chosen through biological rationale or to simplify the modelling process.

In previous comparative studies, we have demonstrated that plant growth form is a predictor of the *potential* magnitude of transient boom or bust, across species and populations. Surprisingly, trees have similarly high transient potential to weeds and herbs of open habitats (Stott *et al*. 2010a). Here, we find that observed population dynamics are dominated by asymptotic growth rates more so in trees than in other growth forms. This difference is not convincing statistically, but it suggests interesting differences between potential and observed transients, that might be linked to the amplitude and frequency of natural disturbances suffered by species of different growth form. For example, tree populations might have great potential for transient boom but rarely experience relevant disturbances at a population scale. Boom might only be experienced following rare events of hurricanes or forest fires (Tuljapurkar 1989; Hoffmann 1999).

### Contribution of Transients to Net Dynamics

A very different picture emerges when we study the contribution of transients to net stochastic dynamics. It is important to note here that, as asymptotic and transient contributions can have opposing effects on net growth, their contribution is not independent: some of the variance explained is shared between the two sources. Transients contribute on average about 65% of the variation in stochastic growth across simulated trajectories.

The contribution of transients increases with increasing numbers of life stages in the respective matrix models. This result is challenging, because its explanation could be biologically rational, or a mathematical artefact. Biological rationale suggests that more complex life histories, which contain a greater number of life stages with distinct vital rates, might be better able to bounce back from demographic disturbances. If most of those life stages are of low reproductive value however, then disturbances might also yield transient bust if reproductive individuals are selectively removed in bad years: indeed, there is evidence that populations which may exhibit larger boom may also be at risk of larger bust (Stott *et al*. 2010a). This is hard to distinguish from a potential mathematical artefact whereby ‘simplified’ models of life histories with fewer stages, which increase sample sizes per stage at the expense of number of stages, underpredict the population's reactive potential by averaging over peaks and troughs in schedules of survival and fecundity. This effect has been demonstrated in real populations (Tenhumberg, Tyre & Rebarber 2009) while the same study found no evidence that larger numbers of stages overpredict the magnitude of the population's transient dynamics. In other words, the choices made by the modeller, during selection of the matrix dimensions, might mask the contribution of transients to stochastic population dynamics. In compadre, for example, there exist examples of long‐lived trees that are modelled using matrices with only three life stages.

### Contribution of Transients to Absolute Dynamics

The analysis of net stochastic dynamics, while describing the observed trajectories of growth and decline, ignores the strength of antagonistic asymptotic and transient effects with opposite signs (Fig. 1). Our consideration of absolute dynamics allows us to partition stochastic dynamics into exclusive contributions from asymptotic dynamics and transient deviations. By doing this, we find that transients still contribute just less than half (47%) of the absolute dynamic. Here, we find repeated evidence for the importance of life‐history complexity: more life stages means greater influence of transients. As discussed above, this could be a biological phenomenon, or a modelling artefact.

### Plant Populations are Booming

An intriguing result is that the average magnitude of reactivity is positive: plant populations tend to boom in response to natural deviations from stable stage structure. At first sight, this result seems counter‐intuitive to our understanding of stochastic dynamics: the expectation would be that boom and bust should somehow be symmetrical around the ‘mean’ dynamic imposed by the statistical distribution of possible environments. This is apparently not the case. Our result is consistent with the possibility that life histories might be selected to maximize their opportunity to boom in response to typical, or even atypical, environmental disturbances. This hypothesis deserves more theoretical and empirical investigation. If, for example, asymptotic and transient growth rates are both targets of natural selection, we might find that declining or threatened populations are declining not just because they have a poor fit to their typical environment, but also because they fail to bounce back from typical demographic disturbances.

Another possible explanation, for the general propensity to boom, stems from the observation that plant population growth rates are distributed relatively closely to one, which implicitly suggests density regulation. We suggest that density‐regulated populations, when disturbed away from carrying capacity, will win the race for available resources not just by having a ‘fast’ asymptotic attractor growth rate at the disturbed density, but also by having a life‐history structure that has evolved to boom in response to sudden availability of resources following disturbances. We recommend the development of further new theory on the interplay between disturbance, density dependence, and asymptotic and transient dynamics.

Stochastic fluctuations in rates of increase are widely accepted to be ‘bad for’ population growth and fitness (except in special circumstances), because multiplicative declines are harder to recoup than multiplicative increases. This thinking has yielded the demographic buffering hypothesis (Pfister 1998), which proposes that selection should favour the reduction in variance in vital rates which contribute most to fitness. Ellis & Crone (2013) theorized that transients could buffer stochastic fluctuations in growth rate if they tended to correlate negatively with fluctuations in stable growth rates. Our observation that transient dynamics consistently amplify population growth regardless of asymptotic growth rate would suggest this is not the case. We note, however, that similar to the demographic buffering hypothesis (Koons *et al*. 2009; Li & Ramula 2015), there is likely considerable variation in the transient properties across life‐history strategies and ecology (Gamelon *et al*. 2014); therefore, we recommend further exploration of the potential role of transient buffering.

### Studying Life‐History Evolution and Population Dynamics in Variable Environments

The usual approach to studying stochastic population dynamics is to model vital rates as random variables and measure long‐term expected growth rates (Benton & Grant 1996; Tuljapurkar, Horvitz & Pascarella 2003). This remains an excellent approach to stochastic evolutionary analysis, but it also suffers from some complications. Stochastic growth rates play out over very long time‐scales and average out the very interesting short‐term interplay between stable and transient growth rates. In stochastic environments, variability may be a mixture of ‘usual’ variation, which might be described using amenable probability distributions, and ‘unusual’ disturbances which can be rare and extreme (Tuljapurkar, Horvitz & Pascarella 2003), defying mathematical representation using means or variances in vital rates. Natural selection of structured life histories will be influenced not just by typical variation around expected vital rates, but also by transient responses to more extreme demographic disturbances. We have observed here that transient dynamics rival stable attractors in their contribution to plant population dynamics and can be long‐lasting. We therefore recommend theoretical work that explores how demographic disturbances (sudden changes to population structure), as well as stochastic vital rates (random variation in projection matrix elements), shape the selection pressures that yield natural life histories.

To date, the analysis of transients has concentrated on stable environments that suffer occasional disturbance, and has tended to examine upper and lower bounds on how big transients can be and how long they will last for. Work in Ellis & Crone (2013), and our analysis here, reveals that transients contribute significantly to stochastic population dynamics, and might help us to understand the evolution of life histories. Perhaps schedules of survival, growth and reproduction evolve to maximize boom, or to exploit transients to buffer against environmental fluctuations. Perhaps variation in parity mode, splits between sexual and clonal reproduction, patterns of senescence and delayed reproduction are life‐history features that emerge from selection on a genotype's ability to bounce back from demographic disturbance. Perhaps endangered species are comprised of those that respond poorly to demographic disturbance. Perhaps population managers could exploit transients to maintain persistence (or cause extinction) of their chosen populations or species (Stott, Hodgson & Townley 2012a). We hope that these analyses prompt further comparative analysis of transient population dynamics in plants and animals, which help to explain the diversity of life histories in nature.

## Data accessibility

compadre data base and phylogeny: available at http://www.compadre-db.org/. R Scripts: included in Appendix S4 (Supporting Information).

## Supporting information


**Figure S1.** Histogram of lambda values for matrices in data sample.

**Appendix S1.** Data used in MCMCglmm analyses.

**Appendix S2.** Code for manipulating compadre data.

**Appendix S3.** Phylogeny used in analyses.

**Appendix S4.** R code for MCMCglmm analyses.

**Appendix S5.** Results of MCMCglmm analyses.
