# Supplementary figures and images for: Transients drive the demographic dynamics of plant populations in variable environments
Source: J Ecol. 2016 Feb 22;104(2):306–14. doi: 10.1111/1365-2745.12528 (PMC4768644; doi:10.1111/1365-2745.12528)

Frequency

300  
200  
100  
0

-3 -2 -1 0 1

$\log(\lambda_{\max})$

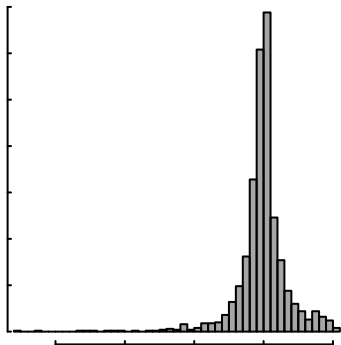

Supplement: Supplementary file 1 — Figure S1. Histogram of lambda values for matrices in data sample. [file JEC-104-306-s001.pdf]
